# Adverse events associated with IL-23 and IL-12/23 inhibitors in the clinical management of psoriasis: a comprehensive pharmacovigilance analysis

**DOI:** 10.1186/s40360-025-00837-y

**Published:** 2025-01-20

**Authors:** Wentao Shi, Ziyi Zhao, Yinghong Zhai, Xiaofei Ye, Feng Xu

**Affiliations:** 1 Clinical Research Unit, Shanghai Ninth People’s Hospital, Shanghai Jiao Tong University School of Medicine, Shanghai, China; 2 Rehabilitation Center, The First Rehabilitation Hospital of Shanghai, Shanghai, China; 3 Department of Health Statistics, Naval Medical University, Shanghai, China

**Keywords:** IL-23 and IL-12/23 inhibitors, Disproportionality analysis, Pharmacovigilance

## Abstract

**Background:**

Interleukin-23 (IL-23) inhibitors and the IL-12/23 inhibitor ustekinumab constitute a pivotal class of therapeutic agents employed in the clinical management of Psoriasis, a chronic immune-mediated skin disorder. Notwithstanding their therapeutic efficacy, concerns have arisen due to the emergence of multiple adverse events (AEs) associated with their usage. This study aims to provide a comprehensive examination of the distribution and characteristics of these AEs concerning IL-23 and IL-12/23 inhibitors, with a specific focus on guselkumab, tildrakizumab, risankizumab, and ustekinumab.

**Methods:**

In this research endeavor, we conducted an extensive analysis of data extracted from the FDA Adverse Event Reporting System (FAERS), spanning the timeframe from January 1, 2014, to September 30, 2022. To identify potential signals of AEs, we rigorously applied disproportionality analysis, utilizing both reporting odds ratio (ROR) and information component (IC) metrics. A signal was considered present when the lower limit of the 95% confidence interval (CI) for ROR (ROR025) exceeded one or when IC (IC025) surpassed zero, with a minimum requirement of three or more reported cases.

**Results:**

Our investigation encompassed a substantial dataset, comprising a total of 41,408,408 reports detailing drug-AE associations and involving 13,271,168 individuals. Among these, 704, 13,164, and 11,399 patients were identified as users of the IL-23 inhibitors tildrakizumab, guselkumab, and risankizumab, respectively, while 62,853 patients were identified as users of the IL-12/23 inhibitor ustekinumab. The analysis revealed the presence of 8, 20, 107, and 115 signals for these respective drugs. Significantly, the System Organ Class (SOC) exhibiting the highest incidence was “infections and infestations,” with documented occurrences in tildrakizumab (6/8), guselkumab (5/20), ustekinumab (50/107), and risankizumab (25/115).

**Conclusion:**

Our pharmacovigilance analysis has brought to light a substantial frequency of AEs linked to IL-23 and IL-12/23 inhibitors. These findings underscore the pivotal role of IL-23 and IL-12/23 inhibitors in modulating immune function and raise concerns regarding their potential to heighten susceptibility to infections and malignancies. However, limitations inherent to the FAERS database, including underreporting, lack of denominator data, potential duplicate records, and inability to confirm causality, should be acknowledged of particular significance is risankizumab, which, despite having fewer reported cases and a later market introduction compared to ustekinumab, exhibited a higher incidence of AEs. These results emphasize the necessity for ongoing vigilance, further investigation, and a reevaluation of the safety profile of IL-23 and IL-12/23 inhibitors in the clinical management of Psoriasis.

**Supplementary information:**

The online version contains supplementary material available at 10.1186/s40360-025-00837-y.

## Background

Interleukin-23 (IL-23) is a pivotal cytokine with a critical role in immune system regulation. This protein molecule is produced by immune cells and serves as a key modulator of immune responses. Belonging to the interleukin-12 cytokine family, IL-23 is intricately involved in orchestrating immune responses to infections and inflammatory processes. IL-23 has been implicated in the pathogenesis of various autoimmune and inflammatory diseases, including but not limited to rheumatoid arthritis, psoriasis, inflammatory bowel disease, and multiple sclerosis [1]. Elevated IL-23 levels have been detected in individuals afflicted with these conditions, hinting at its potential involvement in disease chronicity through the induction and maintenance of T helper (Th) 17 cells, Th22 cells, innate lymphoid cells, and the production of critical effector cytokines, such as interleukin-17, interleukin-22, and tumor necrosis factor (TNF) α [2–5].

To counteract the impact of IL-23, inhibitors have been developed and approved for clinical use, including IL-23 inhibitors (guselkumab, tildrakizumab, and risankizumab), which specifically target the p19 subunit of IL-23, and the IL-12/23 inhibitor ustekinumab, which targets the shared p40 subunit of both IL-12 and IL-23. These inhibitors are currently under investigation and widely used as treatments for autoimmune and inflammatory diseases [6–8]. Although IL-23 inhibitors represent a novel class of drugs, ustekinumab, as an IL-12/23 inhibitor, exhibits a broader mechanism of action by targeting both IL-12 and IL-23 pathways. This broader mechanism may result in distinct safety and efficacy profiles compared to IL-23-specific inhibitors. However, these drugs are grouped together in this study due to their overlapping clinical applications and shared therapeutic goals.

Both IL-12/23 and IL-23 inhibitors are widely used in the treatment of autoimmune and inflammatory diseases, particularly psoriasis, where they target key cytokines involved in the pathogenesis of the disease. By modulating immune responses, these inhibitors aim to reduce inflammation and improve disease outcomes. Furthermore, their similar indications and mechanisms of immune modulation make it clinically relevant to evaluate their safety profiles collectively, as this can provide a more comprehensive understanding of potential adverse events associated with targeting these pathways.

However, akin to all pharmaceutical agents, both IL-12/23 and IL-23 inhibitors may induce adverse events (AEs). Commonly reported AEs associated with IL-23 inhibitors encompass infections, inflammation, allergic reactions, nausea, vomiting, liver problems, and immune reactions [9]. Similarly, ustekinumab has been associated with infections and malignancies, likely due to its broader immunosuppressive effects. Nevertheless, the sole systematic approach for detecting adverse drug reactions relies on the utilization of post-marketing spontaneous reporting systems.

While a study has examined the link between IL-23 inhibitors and cerebrovascular accidents, a comprehensive investigation into other potential adverse reactions remains absent [10]. The objective of this study is to assess potential signals of IL-23 inhibitors (guselkumab, tildrakizumab, risankizumab) and the IL-12/23 inhibitor ustekinumab using post-marketing surveillance data derived from suspected AEs collected within the World Health Organization (WHO) global database of individual case safety reports (ICSRs), VigiBase. Our aim is to provide critical evidence for informing clinical practice.

## Materials and methods

### Data sources and procedures

The FDA Adverse Event Reporting System (FAERS) database represents a renowned publicly accessible post-marketing safety surveillance repository, which is populated with reports from a spectrum of healthcare professionals, including physicians, pharmacists, nurses, and others, as well as consumers, encompassing patients, family members, lawyers, and manufacturers. In our study, we specifically focused on data sets within the FAERS database, which encompass demographic, drug-related, indication, outcome, reaction, report source, and therapy information. As pivotal variables for our investigation, we selected PRIMARYID and CASEID for key matching purposes. Notably, within the drug-related information, we categorized drugs based on their roles as detailed in the ROLE_COD field of the DRUG file, distinguishing them as primary suspects (PS), secondary suspects (SS), concomitant (C), or interacting (I). For the scope of our study, exclusively primary suspect drugs were considered. We extracted relevant data, including crucial variables such as age, gender, reporter country, drug names, preferred terms (PTs), and patient outcomes, from the FAERS database spanning from January 1, 2014, to September 30, 2022. All adverse events (AEs) were coded in alignment with the Medical Dictionary for Regulatory Activities (MedDRA) version 24.0 in English. Correspondingly, the system organ classes (SOCs) associated with these PTs were identified, with SOCs being conceptually equivalent to the systematic classification employed in other medical contexts.

In this study, we analyzed four drugs: guselkumab, tildrakizumab, risankizumab, and ustekinumab, marketed under the trade names Tremfya, Ilumya, Skyrizi, and Stelara, respectively (Table S1). Although guselkumab, tildrakizumab, and risankizumab are IL-23 inhibitors that specifically target the p19 subunit of IL-23, and ustekinumab is an IL-12/23 inhibitor that targets the shared p40 subunit of IL-12 and IL-23, these drugs were analyzed together due to their overlapping clinical applications and shared therapeutic goals.

While these drugs share similar mechanisms of immune modulation, they exhibit differences in their molecular targets, which may influence their safety profiles and therapeutic effects. All four drugs are widely used in the treatment of autoimmune and inflammatory diseases, particularly psoriasis, where they modulate key cytokines involved in immune responses. By targeting pathways central to the pathogenesis of psoriasis and other inflammatory conditions, these drugs share similar mechanisms of immune modulation, making it clinically relevant to evaluate their safety profiles collectively.

### Methods

In our study, we employed disproportionality analysis [11] to detect adverse reactions, which is a methodology aimed at identifying potential signals of adverse reactions or side effects associated with a specific medication post-approval. In the context of adverse reaction detection, disproportionality analysis typically involves comparing the frequency of adverse reactions reported for a particular drug or group of drugs to the expected frequency based on its usage in the population. We used two disproportional signal detection methods [12, 13] based on the frequency and Bayesian theories—the proportional report odds ratio (ROR) and Bayesian confidence propagation neural network of information components (IC)—to verify the stability of the detected signals. In order to get more stable signals, the shrinkage transformation [14] of ROR and IC is calculated as follows:

$${\text{ROR }} = {\text{ }}\left( {{{\text{N}}_{{\text{Observed}}}} + {\text{ }}0.5} \right)/((({{\text{N}}_{{\text{Drug}}}}*{{\text{N}}_{{\text{Event}}}})/{{\text{N}}_{{\text{Total}}}}){\text{ }} + {\text{ }}0.5)$$

$${\text{RO}}{{\text{R}}_{025}} = {e^{{\text{log}}(ror) - 1.96*\sqrt {\frac{1}{a} + \frac{1}{b} + \frac{1}{c} + \frac{1}{d}} }}$$

$${\text{IC}} = log_2^{({{\text{N}}_{{\text{observed}}}} + 0.5)/((({{\text{N}}_{{\text{Drug}}}}*{{\text{N}}_{{\text{Event}}}})/{{\text{N}}_{{\text{Total}}}}) + 0.5)}$$

$${\text{I}}{{\text{C}}_{025}} = {\text{IC}} - 2*{({{\text{N}}_{{\text{Observed}}}} + 0.5)^{ - 1.5}} - 3.3*{({{\text{N}}_{{\text{Observed}}}} + 0.5)^{ - 0.5}}$$

N_Observed_ is the observed number of records of target drug-AEs, N_Expected_ is the expected number of records of target drug-AEs, N_Drug_ is the total number of records of the target drug, N_Event_ is the total number of records of target AEs, and N_Total_ is the total number of records in the whole database. It is a signal when the lower limit of 95% confidence interval (CI) of ROR (ROR_025_) >1 or IC(IC_025_) > 0, with the number of cases ≥3 at the same time. All data cleaning and statistical analyses were conducted using SAS version 9.4 (SAS Institute, Cary, NC, United States)

## Results

A total of 41,408,408 drug-AE reports were extracted from the FAERS database, covering the period from January 1, 2014, to September 30, 2022, involving 13,271,168 people. Among them, 88,121 individuals had been exposed to IL-23 inhibitors, and, specifically, 70,413, 11,164, 62,853, and 11,399 patients had utilized tildrakizumab, guselkumab, ustekinumab, and risankizumab, respectively. The distribution of clinical characteristics among various drug users is detailed in Table 1. In general, the distribution of each characteristic was similar in IL-23 inhibitors related AEs and all AEs. Notably, among all IL-23 and IL-12/23 inhibitor users, females accounted for a higher proportion than males (50.45% vs. 39.38%) or users of any other drugs. The highest proportion of individuals aged 45 to 64 employed IL-23 and IL-12/23 inhibitors (24.39%). There is a discernible upward trend in the number of reports over the years, with a peak observed in 2020, followed by a gradual decline, primarily attributable to reduced use of ustekinumab. The majority of IL-23 and IL-12/23 inhibitor-related AEs originated from the United States (52,741, 59.85%) and Canada (9,593, 10.89%). Hospitalization (15,875, 18.02%) and other serious medical events (29,734, 33.74%) constituted the most frequently reported outcomes. Over the nine-year study period, 3,786 patients experienced severe outcomes, including death, life-threatening conditions, disability, and congenital anomalies, raising safety concerns for IL-23 and IL-12/23 inhibitors. However, IL-23 inhibitors showed a lower overall proportion of serious adverse events compared to other drugs (4.3% vs. 12.46%), with consistently lower rates for death (2.47% vs. 8.68%), life-threatening conditions (1.15% vs. 2.08%), disability (0.58% vs. 1.46%), and congenital anomalies (0.10% vs. 0.24%).

**Table 1:** Characteristics of patients with IL-23and IL-12/23 inhibitors related AEs

	AEs in other drugs	AEs in IL-23 and IL-12/23 inhibitors	tildrakizumab	guselkumab	ustekinumab	risankizumab
**Total**	13,183,047 (99.34)	88,121 (0.66)	704	13,164	62,853	11,399
**Gender**						
Female	6,684,249 (50.70)	44,458 (50.45)	278	6476	32,002	5702
Male	4,424,113 (33.56)	34,706 (39.38)	301	4931	24,422	5051
Unknown or missing	2,074,685 (15.74)	8957 (10.16)	125	1757	6429	646
**Age (year)**						
<18	463,955 (3.52)	2760 (3.13)	1	276	2481	2
18–44	1,522,963 (11.55)	15,743 (17.87)	52	2219	12,507	965
45–64	2,697,327 (20.46)	21,496 (24.39)	101	3763	15,307	2324
65–74	1,538,113 (11.67)	6908 (7.84)	66	899	4743	1200
≥75	1,297,627 (9.84)	2753 (3.12)	29	386	1800	538
Unknown or missing	5,663,062 (42.96)	38,461 (43.65)	455	5621	26,015	6370
**Year**						
2014	901,452 (6.84)	1722 (1.95)	0	0	1722	0
2015	1,314,841 (9.97)	5064 (5.75)	0	0	5064	0
2016	1,300,632 (9.87)	4253 (4.83)	0	0	4253	0
2017	1,363,190 (10.34)	6357 (7.21)	0	62	6295	0
2018	1,675,523 (12.71)	9329 (10.59)	1	1192	8135	0
2019	1,715,174 (13.01)	12,122 (13.76)	94	2149	9533	346
2020	1,738,052 (13.18)	19,317 (21.92)	166	2616	14,514	2021
2021	1,845,210 (14.00)	15,178 (17.22)	247	3306	7301	4324
2022 (Q1–Q3)	1,328,973 (10.08)	14,779 (16.77)	196	3839	6036	4708
**Reporter Country**						
Canada	579,809 (4.40)	9593 (10.89)	12	496	8562	523
Germany	287,063 (2.18)	1790 (2.03)	16	277	1307	190
France	361,387 (2.74)	1323 (1.50)	1	135	1148	39
Great Britain	438,450 (3.33)	7020 (7.97)	15	177	6674	154
Italy	192,930 (1.46)	372 (0.42)	4	33	323	12
Japan	411,244 (3.12)	1460 (1.66)	13	321	1010	116
Netherlands	95,841 (0.73)	230 (0.26)	0	20	210	0
Other countries	1,935,431 (14.68)	13,592 (15.42)	70	681	8515	4326
United States	8,880,892 (67.37)	52,741 (59.85)	573	11,024	35,104	6039
**Outcome**						
Death	1,144,160 (8.68)	2174 (2.47)	20	209	1325	620
Life-threatening	274,309 (2.08)	1010 (1.15)	9	98	829	74
Disability	192,526 (1.46)	513 (0.58)	2	57	377	77
Congenital anomaly	31,452 (0.24)	89 (0.10)	1	0	62	26
Hospitalization	2,343,941 (17.78)	15,875 (18.02)	94	1019	11,573	3189
Other serious	3,649,745 (27.69)	29,734 (33.74)	172	2378	22,173	5010
Required interventio	6873 (0.05)	37 (0.04)	0	2	30	5
Unknown or missing	5,540,041 (42.02)	38,689 (43.90)	406	9401	26,484	2398

**Fig. 1:**
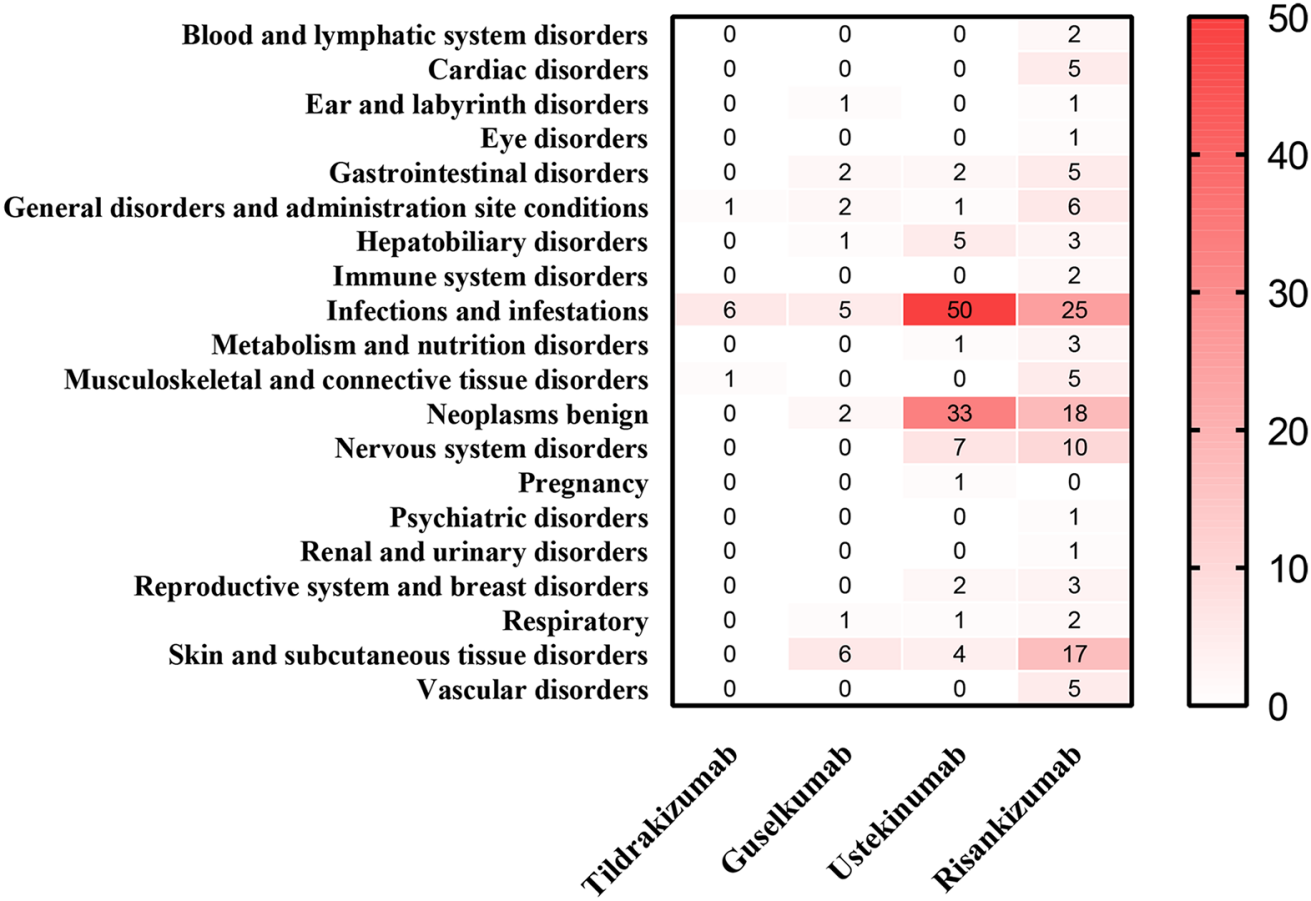
Heat map of adverse event signals of IL-23 and IL-12/23 inhibitors according to SOC. The color intensity corresponds to the quantity, with deeper red indicating higher values

According to the detection conditions of AE signals, tildrakizumab, guselkumab, ustekinumab and risankizumab obtained 8, 20, 107 and 115 signals respectively. The potential adverse drug reaction corresponds to its SOC based on MedDRA are shown in Fig. 1. Table 2 describe the signal between IL-23/IL-12/23 inhibitors and AEs in FAERS database quantified by ROR_025_ and IC_025_. As evidenced in the results, the top 5 most robust signals at the Preferred Term (PT) level were Ovarian germ cell embryonal carcinoma stage II (N = 7, ROR025 = 5.21, IC025 = 2.18), Tuberculosis of peripheral lymph nodes (N = 7, ROR025 = 5.14, IC025 = 2.16), Renal cell carcinoma stage I (N = 9, ROR025 = 4.17, IC025 = 1.85), Viral pericarditis (N = 14, ROR025 = 4.04, IC025 = 1.85), and Alcoholic pancreatitis (N = 13, ROR025 = 4.02, IC025 = 1.83). Additionally, the top 5 most frequently reported AEs associated with IL-23 and IL-12/23 inhibitors were Erythrodermic psoriasis (N = 66, ROR025 = 1.66, IC025 = 0.67), Alcoholic liver disease (N = 23, ROR025 = 2.48, IC025 = 1.19), Cirrhosis alcoholic (N = 20, ROR025 = 1.43, IC025 = 0.39), Abdominal wall abscess (N = 17, ROR025 = 1.22, IC025 = 0.16), and Pityriasis rubra pilaris (N = 16, ROR025 = 4.00, IC025 = 1.85). It is worth noting that all of these signals were observed in ustekinumab. Furthermore, Erythrodermic psoriasis was identified in both guselkumab and risankizumab, and viral pericarditis and Erythrodermic psoriasis were associated with guselkumab.

**Table 2:** Association between IL-23and IL-12/23 inhibitors and AEs in FAERS database at the PTs level

SOC	PT	N	ROR_025_	IC_025_
Gastrointestinal disorders	Alcoholic pancreatitis	13	4.02	1.83
Gastrointestinal disorders	Omental infarction	4	2.31	0.88
General disorders and administration site conditions	Oedema due to cardiac disease	5	2.12	0.79
Hepatobiliary disorders	Alcoholic liver disease	23	2.48	1.19
Hepatobiliary disorders	Cirrhosis alcoholic	20	1.43	0.39
Immune system disorders	Allergy to surgical sutures	3	1.38	0.04
Infections and infestations	Tuberculosis of peripheral lymph nodes	7	5.14	2.16
Infections and infestations	Viral pericarditis	14	4.04	1.85
Infections and infestations	External ear cellulitis	11	2.79	1.30
Infections and infestations	Periumbilical abscess	5	2.67	1.12
Infections and infestations	Perichondritis	7	2.61	1.14
Infections and infestations	Keratitis viral	4	2.61	1.09
Infections and infestations	Mucocutaneous candidiasis	6	2.56	1.09
Infections and infestations	Sporotrichosis	6	2.54	1.08
Infections and infestations	Plasmodium malariae infection	3	1.83	0.57
Infections and infestations	Thyroglossal cyst infection	3	1.82	0.51
Infections and infestations	Bartholin’s abscess	5	1.81	0.56
Infections and infestations	Leptospirosis	4	1.76	0.47
Infections and infestations	Norovirus infection	8	1.72	0.57
Infections and infestations	Moraxella infection	8	1.67	0.52
Infections and infestations	Shigella infection	4	1.61	0.34
Infections and infestations	Burkholderia pseudomallei infection	4	1.59	0.32
Infections and infestations	Omphalitis	11	1.55	0.46
Infections and infestations	Systemic viral infection	3	1.51	0.17
Infections and infestations	Viral hepatitis carrier	3	1.48	0.14
Infections and infestations	Rubella	5	1.46	0.25
Infections and infestations	Infective spondylitis	8	1.46	0.33
Infections and infestations	Tick-borne viral encephalitis	4	1.44	0.18
Infections and infestations	Ovarian abscess	5	1.41	0.20
Infections and infestations	Paracoccidioides infection	3	1.41	0.07
Infections and infestations	Secondary syphilis	3	1.38	0.04
Infections and infestations	Testicular abscess	4	1.37	0.10
Infections and infestations	Genital herpes simplex	7	1.34	0.19
Infections and infestations	Anal infection	12	1.34	0.25
Infections and infestations	Corneal infection	6	1.23	0.04
Infections and infestations	Abdominal wall abscess	17	1.22	0.16
Infections and infestations	Gastritis bacterial	15	1.14	0.05
Metabolism and nutrition disorders	Lipid metabolism disorder	9	1.46	0.35
Musculoskeletal and connective tissue disorders	Calcification of muscle	4	1.41	0.15
Musculoskeletal and connective tissue disorders	Bone hypertrophy	5	1.25	0.03
Neoplasms benign, malignant and unspecified (incl cysts and polyps)	Ovarian germ cell embryonal carcinoma stage II	7	5.21	2.18
Neoplasms benign, malignant and unspecified (incl cysts and polyps)	Renal cell carcinoma stage I	9	4.17	1.85
Neoplasms benign, malignant and unspecified (incl cysts and polyps)	Papillary cystadenoma lymphomatosum	8	3.61	1.63
Neoplasms benign, malignant and unspecified (incl cysts and polyps)	Tongue carcinoma stage 0	5	3.41	1.51
Neoplasms benign, malignant and unspecified (incl cysts and polyps)	Intestinal adenocarcinoma	13	3.21	1.51
Neoplasms benign, malignant and unspecified (incl cysts and polyps)	Transitional cell carcinoma recurrent	5	3.19	1.40
Neoplasms benign, malignant and unspecified (incl cysts and polyps)	Endometrial adenoma	4	2.70	1.23
Neoplasms benign, malignant and unspecified (incl cysts and polyps)	Giant cell tumour of tendon sheath	4	2.33	0.89
Neoplasms benign, malignant and unspecified (incl cysts and polyps)	Testicular seminoma (pure) stage I	5	2.15	0.81
Neoplasms benign, malignant and unspecified (incl cysts and polyps)	Small intestine adenocarcinoma	12	2.12	0.92
Neoplasms benign, malignant and unspecified (incl cysts and polyps)	B-cell lymphoma stage I	4	2.10	0.73
Neoplasms benign, malignant and unspecified (incl cysts and polyps)	Lymphangioma	6	1.96	0.70
Neoplasms benign, malignant and unspecified (incl cysts and polyps)	Chronic lymphocytic leukaemia stage 0	3	1.78	0.46
Neoplasms benign, malignant and unspecified (incl cysts and polyps)	Biliary cancer metastatic	3	1.65	0.32
Neoplasms benign, malignant and unspecified (incl cysts and polyps)	Pleomorphic adenoma	4	1.64	0.36
Neoplasms benign, malignant and unspecified (incl cysts and polyps)	Ependymoma	8	1.54	0.41
Neoplasms benign, malignant and unspecified (incl cysts and polyps)	Ewing’s sarcoma	9	1.54	0.42
Neoplasms benign, malignant and unspecified (incl cysts and polyps)	Dermatofibrosarcoma protuberans	5	1.53	0.32
Neoplasms benign, malignant and unspecified (incl cysts and polyps)	Laryngeal squamous cell carcinoma	5	1.45	0.24
Neoplasms benign, malignant and unspecified (incl cysts and polyps)	Choroid melanoma	4	1.41	0.14
Neoplasms benign, malignant and unspecified (incl cysts and polyps)	Laryngeal neoplasm	5	1.36	0.14
Neoplasms benign, malignant and unspecified (incl cysts and polyps)	Small cell lung cancer metastatic	9	1.36	0.24
Nervous system disorders	Notalgia paraesthetica	5	3.48	1.55
Nervous system disorders	Reversible ischaemic neurological deficit	7	3.15	1.41
Nervous system disorders	Basilar artery aneurysm	4	2.03	0.68
Nervous system disorders	Wernicke-Korsakoff syndrome	3	1.58	0.25
Nervous system disorders	Motor neurone disease	12	1.44	0.36
Nervous system disorders	Dural arteriovenous fistula	4	1.34	0.08
Reproductive system and breast disorders	Breast ulceration	6	1.59	0.40
Respiratory, thoracic and mediastinal disorders	Pneumonia lipoid	5	1.25	0.02
Skin and subcutaneous tissue disorders	Pityriasis rubra pilaris	16	4.00	1.85
Skin and subcutaneous tissue disorders	Rhinophyma	4	2.67	1.15
Skin and subcutaneous tissue disorders	Erythrodermic psoriasis	66	1.66	0.67
Skin and subcutaneous tissue disorders	Chronic actinic dermatitis	3	1.59	0.26
Skin and subcutaneous tissue disorders	Eosinophilic cellulitis	4	1.47	0.20
Skin and subcutaneous tissue disorders	Eczema nummular	8	1.22	0.07
Vascular disorders	Thromboangiitis obliterans	4	1.96	0.62
Vascular disorders	Iliac artery disease	3	1.79	0.47

**Fig. 2:**
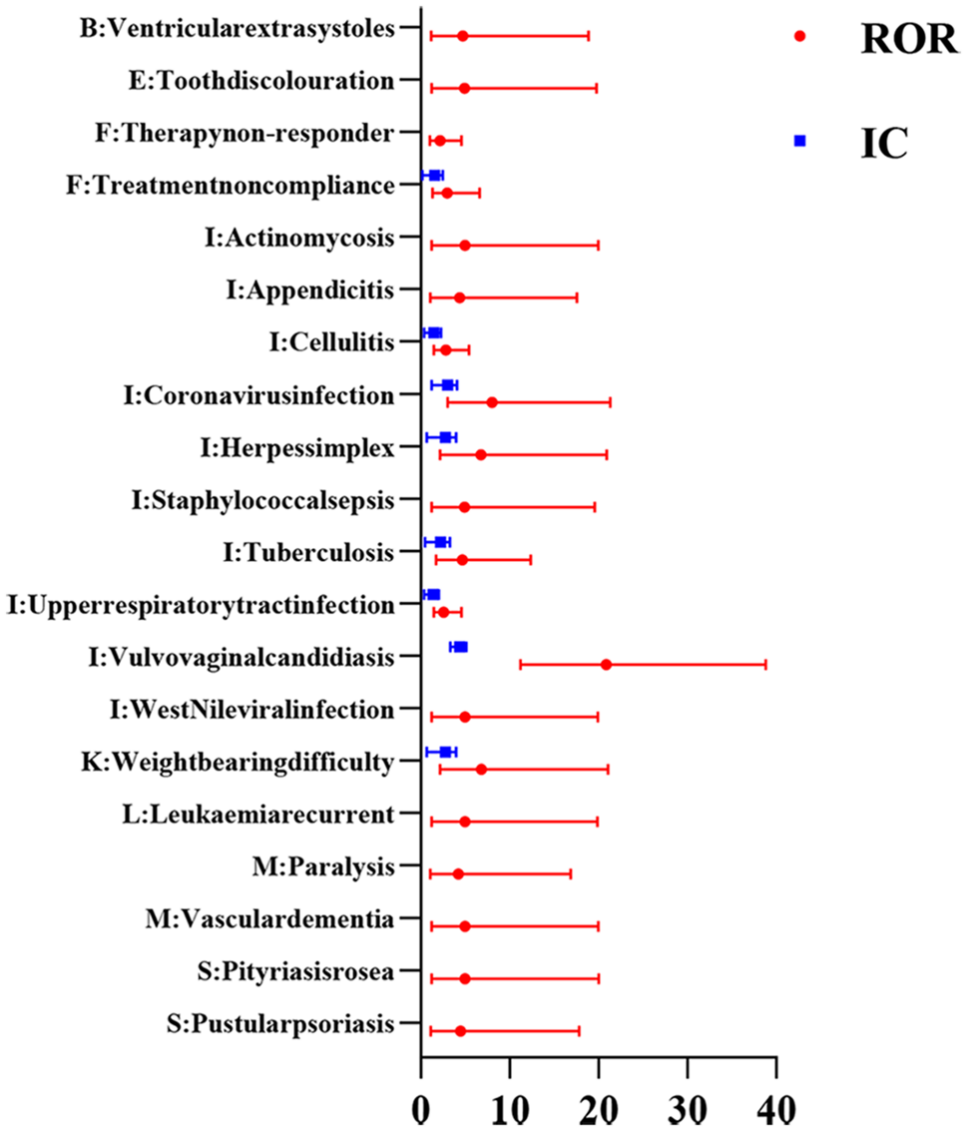
All signals of tildrakizumab at the PT level. The red dots represent the ROR with 95% confidence intervals, while the blue squares represent the IC with 95% confidence intervals. A signal is considered significant when the lower limit of the 95% confidence interval of ROR (ROR025) exceeds 1 or IC (IC025) exceeds 0, with the number of cases being ≥ 3. A: Blood and lymphatic system disorders; B: Cardiac disorders; C: Ear and labyrinth disorders; D: Eye disorders; E: Gastrointestinal disorders; F: General disorders and administration site conditions; G: Hepatobiliary disorders; H: Immune system disorders; I: Infections and infestations; J: Metabolism and nutrition disorders; K: Musculoskeletal and connective tissue disorders; L: Neoplasms benign; malignant and unspecified; M: Nervous system disorders; N: Pregnancy, puerperium and perinatal conditions; O: Psychiatric disorders; P: Renal and urinary disorders; Q: Reproductive system and breast disorders; R: Respiratory, thoracic and mediastinal disorders; S: Skin and subcutaneous tissue disorders; T: Vascular disorders respectively

**Fig. 3:**
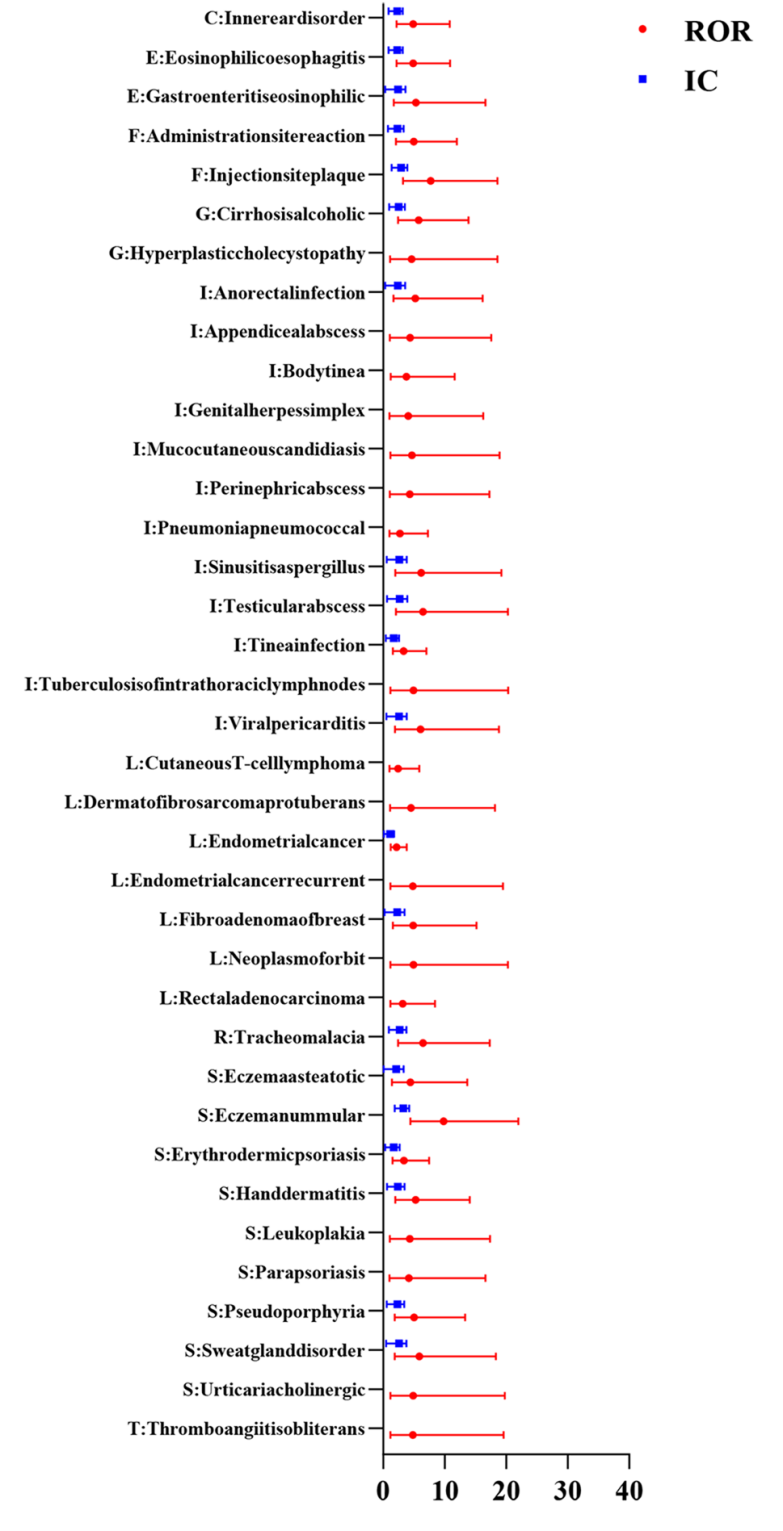
All signals of guselkumab at the PT level. The red dots represent the ROR with 95% confidence intervals, while the blue squares represent the IC with 95% confidence intervals. A signal is considered significant when the lower limit of the 95% confidence interval of ROR (ROR025) exceeds 1 or IC (IC025) exceeds 0, with the number of cases being ≥ 3. A: Blood and lymphatic system disorders; B: Cardiac disorders; C: Ear and labyrinth disorders; D: Eye disorders; E: Gastrointestinal disorders; F: General disorders and administration site conditions; G: Hepatobiliary disorders; H: Immune system disorders; I: Infections and infestations; J: Metabolism and nutrition disorders; K: Musculoskeletal and connective tissue disorders; L: Neoplasms benign; malignant and unspecified; M: Nervous system disorders; N: Pregnancy, puerperium and perinatal conditions; O: Psychiatric disorders; P: Renal and urinary disorders; Q: Reproductive system and breast disorders; R: Respiratory, thoracic and mediastinal disorders; S: Skin and subcutaneous tissue disorders; T: Vascular disorders respectively

**Fig. 4:**
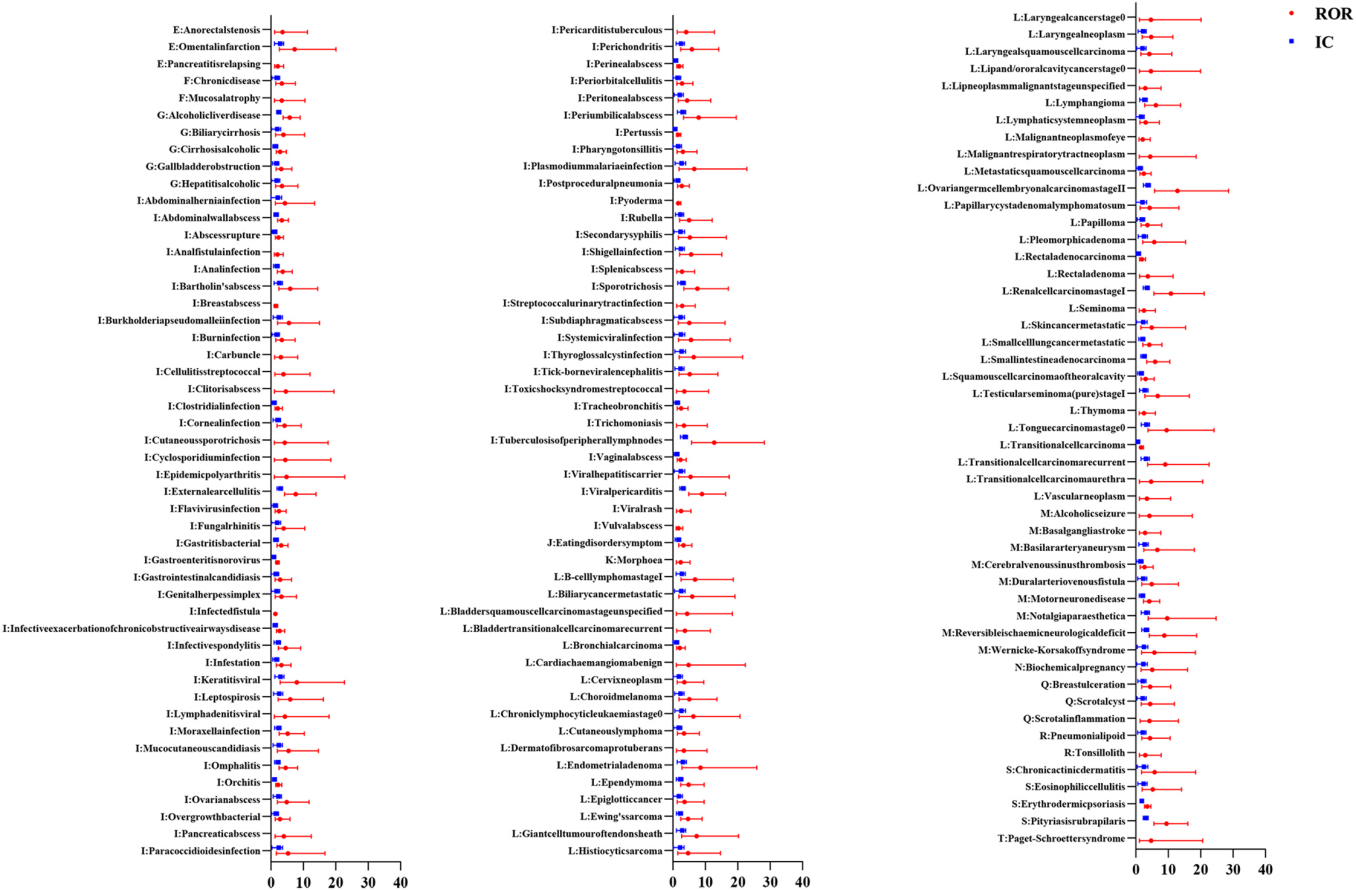
All signals of ustekinumab at the PT level. The red dots represent the ROR with 95% confidence intervals, while the blue squares represent the IC with 95% confidence intervals. A signal is considered significant when the lower limit of the 95% confidence interval of ROR (ROR025) exceeds 1 or IC (IC025) exceeds 0, with the number of cases being ≥ 3. A: Blood and lymphatic system disorders; B: Cardiac disorders; C: Ear and labyrinth disorders; D: Eye disorders; E: Gastrointestinal disorders; F: General disorders and administration site conditions; G: Hepatobiliary disorders; H: Immune system disorders; I: Infections and infestations; J: Metabolism and nutrition disorders; K: Musculoskeletal and connective tissue disorders; L: Neoplasms benign; malignant and unspecified; M: Nervous system disorders; N: Pregnancy, puerperium and perinatal conditions; O: Psychiatric disorders; P: Renal and urinary disorders; Q: Reproductive system and breast disorders; R: Respiratory, thoracic and mediastinal disorders; S: Skin and subcutaneous tissue disorders; T: Vascular disorders respectively

**Fig. 5:**
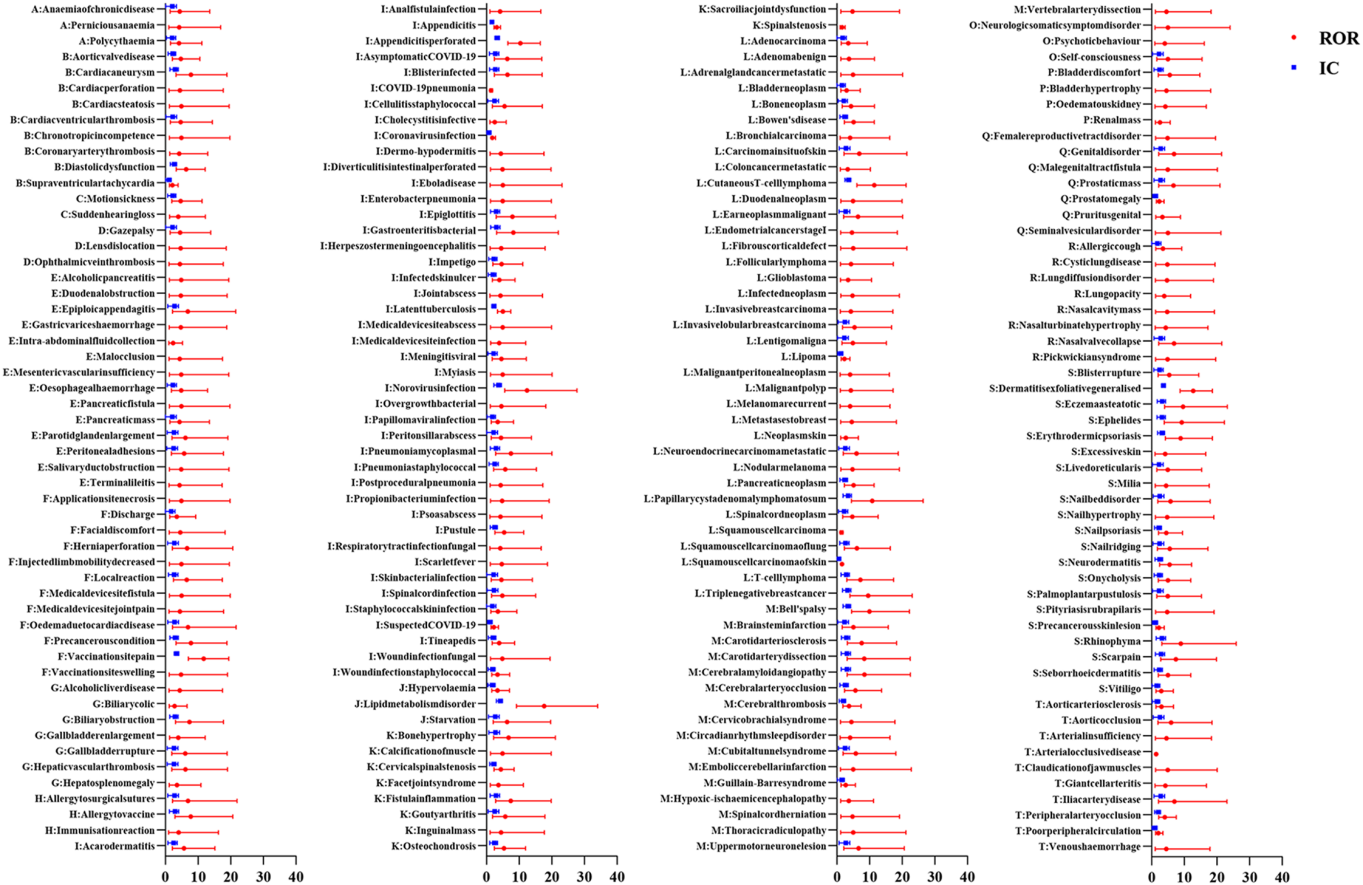
All signals of risankizumab at the PT level. The red dots represent the ROR with 95% confidence intervals, while the blue squares represent the IC with 95% confidence intervals. A signal is considered significant when the lower limit of the 95% confidence interval of ROR (ROR025) exceeds 1 or IC (IC025) exceeds 0, with the number of cases being ≥ 3. A: Blood and lymphatic system disorders; B: Cardiac disorders; C: Ear and labyrinth disorders; D: Eye disorders; E: Gastrointestinal disorders; F: General disorders and administration site conditions; G: Hepatobiliary disorders; H: Immune system disorders; I: Infections and infestations; J: Metabolism and nutrition disorders; K: Musculoskeletal and connective tissue disorders; L: Neoplasms benign; malignant and unspecified; M: Nervous system disorders; N: Pregnancy, puerperium and perinatal conditions; O: Psychiatric disorders; P: Renal and urinary disorders; Q: Reproductive system and breast disorders; R: Respiratory, thoracic and mediastinal disorders; S: Skin and subcutaneous tissue disorders; T: Vascular disorders respectively

Figures 2, 3, 4, 5 show the hierarchical analysis of four drugs. The top three strongest signal at the PT level for tildrakizumab were Vulvovaginal candidiasis (N = 10, ROR_025_ = 11.23, IC_025_ = 3.31), Coronavirus infection (N = 4, ROR_025_ = 3.01, IC_025_ = 1.24), Weight bearing difficulty (N = 3, ROR_025_ = 2.19, IC_025_ = 0.7). Similarly, the top three strongest signal at the PT level for guselkumab were Eczema nummular (N = 6, ROR_025_ = 4.41, IC_025_ = 1.88), Injection site plaque (N = 5, ROR_025_ = 3.2, IC_025_ = 1.38), Tracheomalacia (N = 4, ROR_025_ = 2.43, IC_025_ = 0.93). For ustekinumab, the top three most robust signals at the PT level were Ovarian germ cell embryonal carcinoma stage II (N = 7, ROR025 = 5.78, IC025 = 2.35), Tuberculosis of peripheral lymph nodes (N = 7, ROR025 = 5.74, IC025 = 2.33), and Pityriasis rubra pilaris (N = 14, ROR025 = 5.56, IC025 = 2.31). Lastly, the top three most robust signals at the PT level for risankizumab were Lipid metabolism disorder (N = 9, ROR025 = 9.15, IC025 = 3), Dermatitis exfoliative generalized (N = 27, ROR025 = 8.75, IC025 = 3.03), and Vaccination site pain (N = 15, ROR025 = 7.06, IC025 = 2.68).

## Discussion

Currently, only a limited number of clinical trials and post-market case reports [15, 16] have provided insights into the adverse drug reactions (ADRs) associated with IL-23 and IL-12/23 inhibitors. To the best of our knowledge, this represents the most extensive and comprehensive pharmacovigilance study pertaining to IL-23 and IL-12/23 inhibitors, drawing upon the vast FAERS database. In this investigation, we harnessed disproportionality analysis (utilizing ROR, a frequency-based approach, and IC, a Bayesian-based method) to unearth potential adverse signals linked to IL-23 and IL-12/23 inhibitors. These two distinct statistical methodologies, working in tandem, provide a rigorous, swift, and quantitative means of elucidating the relationship between the target drug and adverse events. Our study contributes a more refined dataset on IL-23 and IL-12/23 inhibitors. In summary, our research unveiled several key findings.

Ustekinumab was the earliest IL-12/23 inhibitor which was approved by FDA in 2009 and it was widely used to cure psoriasis. In nearly nine years of FAERS data, 62,853 people have reported ustekinumab and 107 signals were found in this study. risankizumab was approved in 2019 and the therapeutic effect is significantly better than that of Ustekinumab [17, 18]. But we have detected 115 signals, although only if 11,399 people report by September 2022. The AE number of risankizumab even more than ustekinumab. Therefore, we need to pay special attention to the adverse reactions of risankizumab.

IL-12/23 and IL-23 inhibitors are considered relatively safe treatment options in the management of their indicated diseases [19], such as psoriasis and inflammatory bowel disease. However, Table 2 provides a comprehensive list of adverse events (AEs) reported in real-life patients. It is important to note that many of these AEs are not directly related to the drugs themselves but rather to the underlying diseases or their comorbidities. For example, conditions such as alcoholism and depression are more frequently observed in psoriasis patients, reflecting the disease burden rather than the treatment. Similarly, certain AEs, such as secondary syphilis or viral hepatitis carrier status, are not associated with either the treatment or the diseases being treated. To illustrate, the incidence of small intestine carcinoma in our database was 12 cases out of 88,121 (0.014%), which is significantly lower than the general population incidence in the United States (0.025%) as reported in epidemiological studies [20]. This discrepancy could be attributed to underreporting in the general population or biases inherent in spontaneous reporting systems like FAERS, such as incomplete data or selective reporting. Nevertheless, such findings warrant attention and highlight the need for future research to better understand and monitor these rare but serious events, ensuring improved safety evaluations and patient outcomes.

IL-23 inhibitors are widely used in immune-mediated diseases, such as psoriasis, by regulating immunity and alleviating inflammation. However, these treatments may paradoxically aggravate inflammation in pre-existing conditions or even trigger new inflammatory diseases, a phenomenon known as paradoxical reactions [21–23]. IL-23 and IL-12/23 inhibitors can cause excessive α-IFN, and then induce overexpression of chemokine receptor 3 to allow T cells to migrate to the skin or any other inflamed tissue, leading to a paradoxical reaction ultimately [1, 3, 9, 24]. There are many paradoxical reactions in this study, including Erythrodermic psoriasis, Palmoplantar pustulosis, Eczema asteatotic, Nail psoriasis etc. In some cases, it is difficult to distinguish between insufficient efficacy in treating the disease and paradoxical reaction. When paradoxical reactions lead to serious consequences, the drug should be promptly stopped and replaced with other drugs to treat the primary disease. It is recommended to closely monitor patients receiving treatment with IL-23 and IL-12/23 inhibitors in the clinical setting, especially those with relatively deficient treatment regimens for primary diseases. Early identification and treatment of these paradoxical reactions is very important.

IL-23 inhibitors had the potential to impair immune function resulting in a risk of infection as with any immune modulating agent. The “infections and infestations” has the highest incidence of SOC in tildrakizumab (6/8), guselkumab (5/20), ustekinumab (50/107), risankizumab (25/115). Infection is the most common adverse reaction as stated in the instructions and Some people have serious infections while taking IL-23 and IL-12/23 inhibitors including diverticulitis, cellulitis, appendicitis, cholecystitis, sepsis, gastroenteritis, osteomyelitis, viral infections and urinary tract infections etc. Some people had to be hospitalized for treatment of their infection [25]. Instruct patients to seek medical help if signs or symptoms of clinically important chronic or acute infection occur. If a patient develops a clinically important or serious infection or is not responding to standard therapy, monitor the patient closely and consider discontinuation of IL-23 and IL-12/23 inhibitors until the infection resolves.

Like infection, IL-23 and IL-12/23 inhibitors may lower the ability of your immune system and may increase your risk for certain types of cancers. The “Neoplasms benign, malignant and unspecified (incl cysts and polyps)” also has high incidence of SOC in guselkumab (2/20), ustekinumab (33/107), risankizumab (18/115). Doctors need to give external attention if patients have ever had any type of cancer while taking medicines. At the same time, patients should tell your doctor if you develop any new skin growths during the treatment with IL-23 and IL-12/23 inhibitors.

Several limitations in our study should also be recognized. Firstly, as a spontaneous reporting system, FAERS has inherent flaws such as underreporting, without denominator, duplicate records, uneven information quality and inability to calculate incidence rates. Although we performed manual correction, there may be records of target drugs that were not included. Secondly, as a retrospective real-world study, causality association of our study could not be confirmed. Thirdly, when a report involves several drugs and/or several adverse events, we took combination of drug–AE pair as the basic unit rather than report, so results from our study may subject bias. Additionally, the FAERS database does not consistently provide detailed information on specific indications, limiting our ability to allocate adverse events (AEs) to respective diseases and necessitating future research to better stratify AEs by indication for more precise safety evaluations. For example, inflammatory bowel disease and psoriasis involve entirely different patient populations and use different dosages of Ustekinumab. Furthermore, while our study provides valuable insights into the safety profiles of IL-23 and IL-12/23 inhibitors, it does not directly address drug survival, a key factor in long-term treatment outcomes. Notably, previous studies [26, 27] have highlighted the superior drug survival rates of IL-23 and IL-12/23 inhibitors compared to IL-17 inhibitors, underscoring their sustained efficacy and tolerability. However, our study is the first pharmacovigilance study on IL-23 and IL-12/23 inhibitors based on more than 20 million records. This makes our conclusion more stable compared with those of other studies.

## Electronic supplementary material

Below is the link to the electronic supplementary material.

Supplementary Material 1

## Data Availability

The dataset supporting the conclusions of this article is available via the public FDA Adverse Event Reporting System database found at https://www.fda.gov/drugs/questions-and-answers-fdas-adverse-event-reporting-system-faers/fda-adverse-event-reporting-system-faers-public-dashboard. The ‘FAERS Public Dashboard’ option was then selected prompting to its home page.
