# Supplementary material for: Adverse events associated with IL-23 and IL-12/23 inhibitors in the clinical management of psoriasis: a comprehensive pharmacovigilance analysis
Source: BMC Pharmacol Toxicol. 2025 Jan 20;26:11. doi: 10.1186/s40360-025-00837-y (PMC11748260; doi:10.1186/s40360-025-00837-y)
Supplement: Supplementary file 1 — Supplementary Material 1 [file 40360_2025_837_MOESM1_ESM.docx]

**Appendices**

# Table S1. Generic names, brand names, approval times, and adverse reactions of IL-23 and IL-12/23 inhibitors

| **Generic name** | **Brand name** | **Approval time** | **Adverse Reactions** |
| --- | --- | --- | --- |
| tildrakizumab | Ilumya | 2018 | Upper respiratory infections,Injection site reactions,Diarrhea |
| guselkumab | Tremfya | 2017 | Upper respiratory infections, Headache, Injection site reactions, Arthralgia, Diarrhea, Gastroenteritis, Tinea infections, Herpes simplex infections |
| ustekinumab | Stelara | 2008 | Nasopharyngitis, upper respiratory tract infection, headache, fatigue, vomiting, injection site erythema, vulvovaginal candidiasis/mycotic infection, bronchitis, pruritus, urinary tract infection, sinusitis |
| risankizumab | Skyrizi | 2019 | Upper respiratory infections, Headache, Fatigue, Injection site reactions, Tinea infections |
